# Identifying barriers and facilitators to primary care practitioners implementing health assessments for people with intellectual disability: a Theoretical Domains Framework-informed scoping review

**DOI:** 10.1186/s43058-024-00579-8

**Published:** 2024-04-16

**Authors:** Paul Caltabiano, Jodie Bailie, Alison Laycock, Bradley Shea, Sally Hall Dykgraaf, Nicholas Lennox, Kanchana Ekanayake, Ross Bailie

**Affiliations:** 1 School of Rural Health, The University of Sydney, Dubbo, Australia; 2 Sydney Medical School, The University of Sydney, Camperdown, Australia; 3 University Centre for Rural Health, The University of Sydney, Lismore, Australia; 4 Centre for Disability Research and Policy, The University of Sydney, Camperdown, Australia; 5 Rural Clinical School, Australian National University, ACT, Canberra, Australia; 6 Queensland Centre for Intellectual and Developmental Disability, Mater Research Institute, University of Queensland, Brisbane, Australia; 7 University of Sydney Library, The University of Sydney, Camperdown, Australia; 8 School of Public Health, The University of Sydney, Camperdown, Australia

**Keywords:** Health assessments, Intellectual disability, Primary care, Implementation, Theoretical domains framework, Scoping review

## Abstract

**Introduction:**

People with intellectual disability experience poorer health outcomes compared with the general population, partly due to the difficulties of accessing preventive care in primary care settings. There is good evidence that structured annual health assessments can enhance quality of care for people with intellectual disability, and their use has become recommended policy in several high-income countries. However, uptake remains low. The Theoretical Domains Framework (TDF) offers a conceptual structure for understanding barriers to implementation and has been usefully applied to inform implementation of health assessments for other high-need groups, but not for people with intellectual disability. We conducted a scoping review of the literature, using the TDF, to identify barriers and facilitators influencing primary care practitioners’ implementation of annual health assessments for people with intellectual disability as part of routine primary care practice.

**Methods:**

This study was conducted according to the JBI methodological approach for scoping reviews. Searches were conducted in Medline (OVID-SP), Embase (OVID-SP), PsycINFO (OVID-SP), CINHAL (EBSCO), Scopus (Elsevier) and Web of Science (Clarivate) for relevant peer-reviewed publications up to May 2023. Screening, full-text review and data extraction were completed by two independent reviewers. Data were extracted and mapped to the TDF to identify relevant barriers and facilitators.

**Results:**

The search yielded 1057 publications, with 21 meeting the inclusion criteria. Mapping data to the TDF, the most frequently identified domains were (a) environmental context and resources, (b) skills, (c) knowledge and (d) emotion. Predominant factors impacting on implementation included practitioners’ lack of awareness about health assessments and their identified benefits; inadequate training and experience by practitioners in the delivery of health assessments for people with intellectual disability; insufficient time to provide health assessments; and practitioner burnout.

**Conclusion:**

Using a theory-informed behavioural framework, our review aids understanding of the barriers and facilitators to improving the implementation of health assessments as part of routine care for people with intellectual disability. However, there is a clear need for further qualitative research to examine the perceptions of primary care practitioners regarding implementation barriers and facilitators to health assessments in general, including views from practitioners who are not currently undertaking health assessments.

**Supplementary Information:**

The online version contains supplementary material available at 10.1186/s43058-024-00579-8.

Contributions to the literature

- Using a theory-informed behavioural framework, this scoping review systematically identifies and categorises barriers and facilitators affecting primary care practitioners’ implementation of structured annual health assessments for people with intellectual disability.
- Barriers and facilitators to implementation were most frequently mapped to the following framework domains: (a) environmental context and resources, (b) skills, (c) knowledge and (d) emotion.
- There is a need for further qualitative research to examine the perceptions of primary care practitioners regarding implementation barriers and facilitators to health assessments in general, and to ensure that the views of primary care practitioners not currently providing health assessments are investigated.

## Introduction

People with intellectual disability experience higher rates of mortality [1] and morbidity [2] compared with the general population. These additional health burdens are present across the life-course and are often ineffectively managed or under-recognised [3]. Inadequate access to preventive care is thought to contribute to inequitable health outcomes for people with intellectual disability [4].

To address these health disparities, structured comprehensive annual health assessments for people with intellectual disability, delivered in primary care settings, have become a feature of health policies in some high-income countries [5, 6]. These assessments are best viewed as a vehicle for improving the delivery of evidence-based preventive care and have been used to target priority population groups, such as people with intellectual disability, the elderly, children and, in Australia, Aboriginal and Torres Strait Islander people.

Multiple publications, including a systematic review that synthesised evidence from 80 studies in the UK [7], have found that health assessments for people with intellectual disability identify new health needs [8, 9], improve the management of existing health needs [10, 11] and enable the provision of health promotion [8, 12, 13]. Crucially, patients with intellectual disability who receive regular health assessments have a lower mortality rate than those who do not [14]. Despite this evidence, uptake of annual health assessments in primary care has been low [9, 15].

The Theoretical Domains Framework (TDF) was initially developed and validated by behavioural scientists to identify behavioural barriers and facilitators related to the implementation of evidence-based recommendations among health professionals [16, 17]. The TDF, which has 14 theoretical domains and 84 constructs derived through a systematic expert consensus process, provides a basis for understanding the broad set of factors that may influence behaviours (Table 1). It has also been used as a framework for synthesising behavioural influences in reviews reporting perceived barriers and facilitators, including: the adoption of prescribing guidelines [18], the de-implementation of low-value care [19], and the treatment and transfer of acute stroke patients in emergency care settings [20].

**Table 1:** TDF Domains and constructs, as espoused by Cane et al. [16]

Domain	Constructs
**Knowledge—**An awareness of the existence of something	Knowledge (including knowledge of condition / scientific rationale); Procedural knowledge; Knowledge of task environment
**Skills—**An ability or proficiency acquired through practice	Skills development; Competence; Ability; Interpersonal skills; Practice; Skill assessment
**Social/Professional Role and Identity—**A coherent set of behaviours and displayed personal qualities of an individual in a social or work setting	Professional identity; Professional role; Social identity; Identity; Professional boundaries; Professional confidence; Group identity; Leadership; Organisational commitment
**Beliefs about Capabilities—**Acceptance of the truth, reality, or validity about an ability, talent, or facility that a person can put to constructive use	Self-confidence; Perceived competence; Self-efficacy; Perceived behavioural control; Beliefs; Self-esteem; Empowerment; Professional confidence
**Optimism—**The confidence that things will happen for the best or that desired goals will be attained	Optimism; Pessimism; Unrealistic optimism; Identity
**Beliefs about Consequences—**Acceptance of the truth, reality, or validity about outcomes of a behaviour in a given situation	Beliefs; Outcome expectancies; Characteristics of outcome expectancies; Anticipated regret; Consequents;
**Reinforcement—**Increasing the probability of a response by arranging a dependent relationship, or contingency, between the response and a given stimulus	Rewards (proximal / distal, valued / not valued, probable / improbable; Incentives; Punishment; Consequents; Reinforcement; Contingencies; Sanctions
**Intentions—**A conscious decision to perform a behaviour or a resolve to act in a certain way	Stability of intentions; Stages of change model; Transtheoretical model and stages of change
**Goals—**Mental representations of outcomes or end states that an individual wants to achieve	Goals (distal / proximal); Goal priority; Goal / target setting; Goals (autonomous / controlled; Action planning; Implementation intention
**Memory, Attention and Decision Processes—**The ability to retain information, focus selectively on aspects of the environment and choose between two or more alternatives	Memory; Attention; Attention control; Decision making; Cognitive overload / tiredness
**Social influences—**Those interpersonal processes that can cause individuals to change their thoughts, feelings, or behaviours	Social pressure; Social norms; Group conformity; Social comparisons; Group norms; Social support; Power; Intergroup conflict; Alienation; Group identity; Modelling
**Emotion—**A complex reaction pattern, involving experiential, behavioural, and physiological elements, by which the individual attempts to deal with a personally significant matter or event	Fear; Anxiety; Affect; Stress; Depression; Positive / negative affect; Burn-out
**Behavioural Regulation—**Anything aimed at managing or changing objectively observed or measured actions	Self-monitoring; Breaking habit; Action planning

In addition, the TDF has been used to examine the uptake of health assessments for targeted population groups, such as for people with autism [21], children [22] and adults with cardiovascular disease [23]. However, to date it has not been used to understand determinants of effective implementation of health assessments for people with intellectual disability.

By assessing the published literature against the TDF, we aimed to identify and categorise barriers and facilitators that influence the implementation of structured health assessments for people with intellectual disability as part of routine practice in primary care. We anticipate that our review findings will contribute to a greater understanding of implementation barriers and facilitators and how they operate to influence practitioner behaviour.

## Methods

Scoping review methodology was selected because our purpose was to systematically identify and characterise the breadth of research that exists around implementation factors, and distinguish the barriers and facilitators to implementation of preventive health assessments [24, 25]. This review drew on methodological guidance for scoping reviews from JBI [26], and was conducted in accordance with a published *a priori* protocol [27]. Reporting was guided by the Preferred Reporting Items for Systematic Reviews and Meta-Analyses Extension for Scoping Reviews (PRISMA-ScR) checklist [28]. Critical appraisal and risk of bias assessment were not conducted, consistent with JBI methodology for scoping reviews.

### Stage 1: research question

The research question was: ‘What are the barriers and facilitators to primary care practitioners implementing comprehensive health assessments as part of routine practice in primary care for people with intellectual disability?’

### Stage 2: relevant literature identification

An initial search of Medline (OVID-SP) and Google Scholar was conducted to identify key publications on the topic and develop a list of search terms. A full search strategy for MEDLINE (OVID-SP) was subsequently developed in consultation with an academic librarian (KE) and research experts in the fields of preventive health assessments, primary care and disability (SHD, NL, RB, JB, BS, AL). The search was then systematically repeated in Medline (OVID-SP), Embase (OVID-SP), PsycINFO (OVID-SP), CINHAL (EBSCO), Scopus (Elsevier) and Web of Science (Clarivate). Database searches were conducted on 1 May 2023. The final search strategy can be found in Additional file 1. Grey literature and theses were not searched.

### Stage 3: study selection

All identified citations were uploaded into COVIDENCE [29], a web-based review platform, and duplicates removed. Following a pilot review, we undertook title and abstract screening and then full-text review using predetermined inclusion and exclusion criteria (Table 2). Two reviewers (PC and JB) independently conducted all stages, with disagreements resolved through discussion.

**Table 2:** Inclusion and exclusion criteria

Inclusion criteria	Exclusion criteria
1. *Population:* People with intellectual disability, defined as permanent decreased intellectual function, present during developmental periods, before age 18. People with cerebral palsy, autism or other neurodevelopmental disorders are only included if they have a co-existing intellectual disability. 2. *Concept:* Barriers and facilitators to implementation of comprehensive health assessments/health checks as identified by clinicians in primary care (GPs and practice nurses). 3. *Context:* General practice / family medicine / primary care, in all countries (i.e. both high and low-income settings). 4. *Types of evidence sources:* Original research from peer-reviewed publications, including quantitative, qualitative, and mixed-method study designs.	1. Full text is not published in English. 2. Full text unavailable. 3. Publication is a report of a research protocol, book, book chapter, thesis, letter to editor, conference abstract, commentary, expert opinion, systematic or narrative review, or practice guidelines.

### Stage 4: data extraction

A data extraction template, developed within COVIDENCE and based on the scoping review template by JBI, was utilised. The template considered the methodological and design characteristics of each publication, study setting, and factors influencing uptake or implementation of health assessments. The data extraction tool underwent a pilot phase using two randomly selected publications. Following refinement through discussion, the tool was updated before application to the remaining publications (Additional file 2). Data extraction was carried out independently by JB and PC.

### Stage 5: data analysis and presentation

As data were extracted, JB and PC independently deductively coded according to the single most relevant TDF domain. To do this, JB and PC read the whole publication, and then line-by-line considered the relevance to the definitions of each domain, attributing the data to the most relevant domain. To guide the data extraction and coding we developed a code book *a priori*. This code book was updated iteratively throughout the data extraction and analysis process by PC and JB. To facilitate consensus for coding extracted data to the most relevant TDF domain, JB and PC articulated their understanding of the coded text (i.e. key meaning) and justified their rationale for selecting the TDF domain by writing notes. JB and PC meet regularly, and resolved through discussion any differences in understanding of the most relevant domain the data should be coded to. Examples of data coded and categorised is provided in Table 3.

**Table 3:** Examples of data coding and categorisation for the first two domains of the TDF

TDF Domain	Example of extracted data coded to TDF domain and then categorised
**Domain 1: Knowledge**	Facilitator: ‘When asked to consider their experience with patients with ID, almost two-thirds of the participating GPs (61%) believed the health of these patients was worse than that of the general population’ [30].
	Facilitator: ‘... the health assessment process was viewed by GPs as a means of improving knowledge and understanding of the wide range of health issues and needs experienced by people with ID’ [30].
	Barrier: ‘[There was a] lack of awareness by GPs of the Medicare funded health assessments [for people with intellectual disability]’ [31].
	Barrier: ‘GPs noted that they do not always label patients with ID with a specific ICPC code in the GP's medical system. The reasons given for this were that some GPs did not know this code...’ [32]
**Domain 2: Skills**	Facilitator: ‘... ongoing efforts are necessary to continually educate GPs to ensure that the health needs of people with learning disabilities are understood’ [33].
	Barrier: ‘... [GPs] simultaneously reported having little practical knowledge of working with individuals with IDs’ [34].

Data coded to TDF domains were analysed in a recursive process that followed the steps of content analysis outlined by Elo and Kyngas [35]. Specifically:

1. PC and JB independently immersed themselves in the extracted data, reading and re-reading publications to get a sense of the whole, primarily to gain a general understanding of the data that had been deductively coded to TDF domains.
2. Within each TDF domain, PC and JB coded data as barriers or facilitators, writing notes and headings describing the content. ‘Barriers’ were defined as behaviours that impeded the implementation of health assessments, and ‘facilitators’ those that promoted health assessments. Examples of coded data categorised as a barrier or facilitator are detailed in Table 3.
3. Building on the initial categorisation of barriers and facilitators, PC and JB developed higher level ‘factors’ that described the barrier/and or facilitator.
4. Through a process of comparison, rereading and revisiting source publications to review context, PC and JB refined the barriers, facilitators, and factors within each TDF domain.

During analysis it became apparent that study participants within some publications perceived the same factor differently. Consequently, a TDF domain could be mapped as both a barrier and a facilitator for the same publication. For example, some practitioners within a publication had known of or were already implementing health assessments for people with intellectual disability, whereas others within the same publications were unaware. Throughout this process, PC and JB conferred to resolve any differences in categorisation or perceptions of relevance. This included reflection sessions between PC and JB, and collaboration with authors RB and AL. To ensure consistency, all authors, drawing on their experience, checked the results against their understanding of how targeted preventive health interventions were implemented in primary care, any access barriers to primary care for people with intellectual disability and the TDF itself.

## Results

### Search results and publication selection

**Fig. 1:**
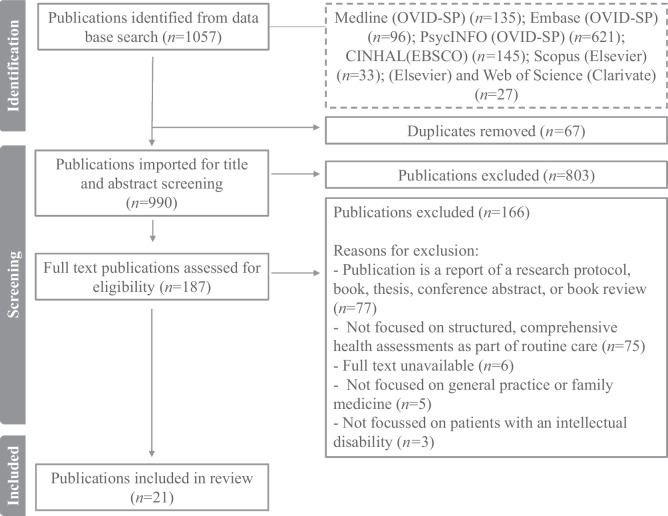
PRISMA-ScR flow diagram

The search yielded 1057 publications. After duplicate removal, title and abstract screening, and full-text review, 21 publications were included as depicted in Fig. 1.

### Characteristics of included studies

The characteristics of the 21 included publications, derived from 20 studies, are presented in Table 4. The majority were qualitative study designs (*n*=12). All included publications were undertaken in one of four high-income countries, presented here in descending order by frequency: United Kingdom (*n*=14) [33, 34, 36–41, 43, 45, 46, 48–50], Australia (*n*=3) [30, 31, 44], Canada (*n*=3) [12, 42, 47], and the Netherlands (*n*=1) [32]. Most were published between 2011 and 2023 (*n*=15), with the remaining (*n*=6) published between 1996 and 2002. The majority did not specify the rurality of the study setting, but five did include regional or rural perspectives [31, 45, 48–50]. Some publications had the primary care practice as the unit of analysis (*n*=8), whereas others included perspectives solely from individual general medical practitioners (GPs) (*n*=7), both GPs and practice nurses (*n*=5), or practice nurses alone (*n*=1). Most publications (*n*=13) were set in primary care practices that were already implementing health assessments. Two publications from one study included people with intellectual disability as part of the research team [48, 49].

**Table 4:** Publication characteristics, including barriers and facilitators mapped to the TDF

First author (year)	Study location; rurality (if stated)	Study design; methods; study dates (if stated)	Study population	Health assessments currently being implemented*	Mapped Domains of the TDF†													
					1	2	3	4	5	6	7	8	9	10	11	12	13	14
Anderson and Jones (2015) [36]	South England, UK	Mixed-methods; Audit and self-assessment; Apr 2014–Mar 2015	33 practices	Yes	●	●						◎		●				〇
Bakker-vanGijssell E, et al. (2017) [32]	The Netherlands	Qualitative; Focus group; Dec 2014–Feb 2015	23 GPs	No	●	●	〇	●		◎	●			●	◎		◎	
Bollard (1999) [33]	UK	Mixed-methods; Interview, survey, and audit; 1997–1998	12 practices, 13 GPs	No	●	●	〇		〇			〇		●	〇		◎	〇
Bond L, et al. (1997) [37]	Gwent, Wales and Gloucestershire, England, UK	Quantitative; Survey; 1994–1995	125 Welsh and 132 English GPs	No			◎								◎	〇	●	
Burton and Walters (2013) [31]	Adelaide, Australia Rural	Qualitative; Interview	3 GPs	Yes	◎	●	〇	〇	◎	●	〇				◎	〇		
Chambers R, et al. (1998) [38]	North Staffordshire, England, UK	Mixed-methods; Focus group, document review, and audit; Mar 1995–May 1996	21 practices	No		●					●				●		●	
Chinn (2020) [39]	London, England, UK	Qualitative; Direct observation and conversation analysis; July 2016–July 2017	32 GP checks, 9 PN checks	Yes				●				〇						
Chinn and Rudall (2021) [40]	London, England, UK	Qualitative; Direct observation and conversation analysis; July 2016–June 2017	8 GPs, 4 PNs	Yes		◎									◎			
Chinn (2022) [41]	England, UK	Qualitative; Direct observation and conversation analysis	6 GPs, 3 PNs	Yes		●									◎			
Durbin J, et al. (2016) [42]	Ontario, Canada	Qualitative; Focus group and document review	2 practices	No		〇					〇	◎	〇	◎	◎		●	〇
Durbin J, et al. (2019) [12]	Ontario, Canada	Quantitative; Audit and survey; July 2013–Aug 2015	147 clinical staff	Yes	◎	●		◎	〇	〇	●				●			
Kerr M, et al. (1996) [43]	Gwent, South Wales, UK	Quantitative; Survey; 1994	126 GPs	No	〇		●			●				●	〇		●	
Lennox N, et al. (2001) [44]	Queensland, Australia	Mixed-methods; Survey, document review audit, and self-assessment	15 GPs completed all components, 45 total	No	〇	●				〇					〇	〇		
Lennox N, et al. (2013) [30]	Greater Brisbane area, Queensland, Australia	Qualitative; Interview; Aug 1998–Sept 2000	46 GPs	Yes	◎	●	●		◎	〇		●			◎			●
Macdonald S, et al. (2018) [34]	Greater Glasgow and Clyde, Scotland, UK	Qualitative; Interview; Mar 2012–Apr 2012	15 PNs	No	〇	●	●	〇	◎						◎		◎	
McConkey R, et al. (2002) [45]	Northern Ireland, UK 35% from rural area	Mixed-methods; Survey	70 GPs	No	◎	●	◎		●	〇		〇			〇		●	
McConkey R, et al. (2015) [46]	Northern Ireland, UK	Quantitative; Audit; 2011/12 2013/14	351 practices	Yes	●			●	●						●		●	
Shooshtari S, et al. (2017) [47]	Manitoba, Canada	Qualitative; Focus group and interview	9 GPs, 3 PNs	Yes		●	◎	〇	〇	〇	●	●			◎	〇	〇	
Walmsley J (2011) [48] and Michell B (2012) [49]	Oxfordshire, England, UK Urban and rural	Qualitative; Interview; Inclusive research	6 practices	Yes	●	●			◎	〇	●	◎		●	◎		〇	
Wigham S, et al. (2022) [50]	UK Regional	Qualitative; Focus group, interview, and survey; June 2021–Dec 2021	7 GPs or PNs	Yes	●	●	〇			〇		〇		●	〇	〇	〇	◎
Total number of publications					13	16	10	7	9	10	7	9	1	7	18	5	12	5

*GP* General Practitioner, *PN* Practice Nurse, *UK* United Kingdom ● = Barrier, 〇 = Facilitator, ◎ = Barrier and Facilitator

† TDF Domains: 1—Knowledge; 2—Skills; 3—Social/Professional Role and Identity; 4—Beliefs about Capabilities; 5—Optimism; 6—Beliefs about Consequences; 7—Reinforcement; 8—Intentions; 9—Goals; 10—Memory, Attention and Decision Processes; 11—Environmental Context and Resources; 12—Social Influence; 13—Emotion; 14—Behavioural Regulation

‡ These publications were collapsed for data extraction as they reported on the same data

### Barriers and facilitators to implementation

In our review of the barriers and facilitators influencing practitioners’ behaviour regarding the implementation of health assessments, data were most frequently coded to the following TDF domains: a) environmental context and resources, b) skills, c) knowledge, and d) emotion. The frequency of each TDF domain is presented in Table 5.

**Table 5:** Barriers and facilitators to implementation identified according to the TDF

TDF domain, number of publications identified and TDF definition	Factors	Practitioners’ perspectives	
		Barriers	Facilitators
**Domain 1: Knowledge (*n*=13)** An awareness of the existence of something.	Level of awareness of potential health outcomes	Lack of awareness of the adverse health outcomes experienced by people with intellectual disability [12, 32, 48] (*n*=3)	Awareness of the adverse health outcomes experienced by people with intellectual disability [12, 30, 43, 44] (*n*=4)
	Level of awareness of existence of assessments	Lack of awareness of the existence of, or what is entailed in, health assessments for patients with intellectual disability [12, 31, 36] (*n*=3)	Awareness of the existence of health assessments for patients with intellectual disability [31, 45] *(n*=2)
	Comprehension of health benefits of assessments	Lack of awareness of the determined health benefits of health assessments [30, 31, 33, 45, 46, 50] (*n*=6)	Awareness of the determined health benefits of health assessments [30, 34] (*n*=2)
	Comprehension of specific codes in clinical information systems and preventive care guidelines	Limited knowledge on specific codes and guidelines, such as those used to identify patients with intellectual disability in their clinical information system [32, 50], and preventive care guidelines [31] (*n*=3)	
**Domain 2: Skills (*n*=16)** An ability or proficiency acquired through practice.	Ability to communicate effectively with patient	Lacking the necessary communications skills or perceiving communication difficulties as a barrier to conducting a health assessment [30, 32, 34, 40, 41, 48] (*n*=6)	Possessing the necessary skills to conduct a health assessment, such as communication [40] and addressing patients with intellectual disability in a respectfully sensitive manner [42] (*n*=2)
	Level of training and experience underpinning work with patient group, including reasonable adjustments Identification of further training needed to improve patient care	Lack of existing training and experience with patients with intellectual disability [31, 32, 34, 45, 47, 48], including understanding what reasonable adjustments are and how to implement them [12, 48, 50] *(n*=8) Identification of recommended areas of training such as further education on provision of care of people with intellectual disability [31–33, 38, 44, 50], specific training on performing health assessments [33, 34, 36], and exposing practitioners to health care needs of people with intellectual disability in their training [31, 47] (*n*=9)	
**Domain 3: Social/Professional Role and Identity (*n*=10)** A coherent set of behaviours and displayed personal qualities of an individual in a social or work setting.	Personal views on role of primary care to deliver health assessments Clarity of role of primary care practitioners in delivery of health assessments	Belief that health assessments should not be delivered by primary care services [37, 43, 45] (*n*=3) Further clarity on requirements of role requested [30, 34] (*n*=2)	Acceptance of primary care provider’s role in delivering health assessments [31–33, 37, 45, 47, 50] (*n*=7)
	Beliefs around who is responsible for follow-up care	Concerns regarding whose responsibility it will be to follow-up on any required actions as a result of assessments [47] (*n*=1)	Acknowledgement of the importance of planning follow-up to the health care assessment (i.e. referral to specialist services if required) [50] (*n*=1)
**Domain 4: Beliefs about Capabilities (*n*=7)** Acceptance of the truth, reality or validity about an ability, talent or facility that a person can put to constructive use.	Level of confidence in ability to perform health assessments	Indicating a low level of self-confidence in one’s own and other practitioners’ abilities to conduct health assessments [12, 32, 39, 46] (*n*=4)	Indicating a high level of self-confidence in one’s own and other practitioners’ abilities to conduct health assessments [12, 31, 34, 47] (*n*=4)
**Domain 5: Optimism (*n*=9)** The confidence that *things will happen for the best* or that desired goals will be attained.	Overall view on health assessments being worthwhile and will improve overall health outcomes	General belief that health assessments are not worthwhile and will not improve the overall health and wellbeing of people with intellectual disability [30, 31, 34, 45, 46, 48] (*n*=6)	General belief that health assessments are worthwhile and will improve the overall health and wellbeing of people with intellectual disability [12, 30, 31, 33, 34, 47, 48] (*n*=7)
**Domain 6: Beliefs about Consequences (*n*=10)** Acceptance of the truth, reality or validity about *outcomes* of a behaviour in a given situation.	Whether health assessments contribute to knowledge and skills for practitioners, support workers and collaboration together		Belief that the process of doing health assessments enhances the knowledge and training of practitioners [12, 32, 47] or support workers [30, 48], and their collaboration together [44, 47] (*n*=6)
	Overall view on effectiveness for health outcomes	Belief that more evidence is still required to indicate the benefits of health assessments [31, 32, 43] (*n*=3)	Belief that health assessments will truly improve objective health outcomes for patients with intellectual disability who utilise them, such as the detection of previously unidentified or preventive health problems and delivery of proactive care [30, 32, 44, 45, 47, 48, 50] (*n*=7)
	Level of benefit for practitioner-patient relationship		Belief that health assessments improve the practitioner–patient relationship [32, 47, 48] (*n*=3)
**Domain 7: Reinforcement (*n*=7)** Increasing the probability of a response by arranging a dependent relationship, or contingency, between the response and a given stimulus.	Adequacy of financial compensation	Belief there is a lack of adequate financial compensation for the time required to prepare for and conduct a health assessment [12, 32, 38, 47, 48] (*n*=5)	Belief there is adequate financial compensation for the time required to prepare for and conduct a health assessment [31, 42] (*n*=2)
**Domain 8: Intentions (*n*=9)** A conscious decision to perform a behaviour or resolve to act in a certain way.	Interest in prioritising heath assessments	Lack of interest in prioritising and providing health assessments to people with intellectual disability [30, 36, 42, 47, 48] (*n*=5)	Invested interest in prioritising and providing health assessments to people with intellectual disability [33, 42, 45, 50], including provision of reasonable adjustments a priority [36, 39, 48, 50] (*n*=7)
**Domain 9: Goals (*n*=1)** Mental representations of outcomes or end-states that an individual wants to achieve.	Willingness to set clear and defined goals		Clear and defined goals to promote uptake of health assessments [42] (*n*=1)
**Domain 10: Memory, Attention and Decision Processes (*n*=7)** The ability to retain information, focus selectively on aspects of the environment and choose between two or more alternatives.	Use of staff reminders		Utilisation of reminders to alert staff of upcoming health assessments [42] (*n*=1)
	Availability of fit-for-purpose patient registry	Insufficient, or inability of, pre-existing patient registry to identify eligible people with intellectual disability [32, 33, 36, 42, 43, 48, 50] (*n*=7)	
**Domain 11: Environmental Context and Resources (*n*=18)** Any circumstances of a person’s situation or environment that discourages or encourages the development of skills and abilities, independence, social competence and adaptive behaviour.	Views on capacity and effectiveness of support workers	General concerns about the capacity of support workers to contribute to the process (i.e. lack of knowledge of relevant medical background or to implement actions arising from health assessments) [30, 31, 40, 41] (*n*=4)	General belief that support workers are useful in the implementation and flow of health assessments [31, 33, 40, 41, 45, 48, 50] (*n*=7)
	Availability of support staff	Unknown, or lack of, support workers [30, 38] or allied health workers and specialists to refer to or seek assistance from [31, 32, 37, 48] (*n*=6)	Others who aren’t primarily in charge of providing the health assessment, including support staff [31, 32, 45] and specialist services [32, 33, 37, 43] (*n*=6)
	Adequacy of resources available to promote the delivery of health assessments Whether enough time is available to perform health assessments	A perceived lack of general resources to support the implementation of health assessments [12, 32, 42, 48] (*n*=4) Inadequate time available or allocated for provision of health assessments [30–32, 34, 38, 42, 47] (*n*=7)	Tools such as the health assessment proformas (e.g. CHAP, Cardiff) [30, 44, 47, 48], ability to access patient histories [31, 34], and electronic formatting of the checks [42, 47] to hasten the process. (*n*=7)
	Patient-related factors that may influence uptake of health assessments	Concern about patient-related factors that act as a deterrent to the uptake of health assessments, such as lack of demand [46], limited access to health care [30], inability to contact [48], and extended length of appointment [42] (*n*=4)	
**Domain 12: Social Influence (*n*=5)** Those interpersonal processes that can cause individuals to change their thoughts, feelings or behaviours.	Perceptions of support to promote the uptake of health assessments		Perceived support to promote the uptake of health assessments from agencies [37, 47], communities [31, 44] or colleagues [50] (*n*=5)
**Domain 13: Emotion (*n*=12)** A complex reaction pattern, involving experiential, behavioural and physiological elements, by which the individual attempts to deal with a personally significant matter or event.	Personal attitudes towards performing health assessments	Generally negative affect towards performing health assessments (i.e. anxiety [33], burnout [34, 37, 38, 43, 45, 46], aversion to checklists [32] (*n*=8)	Generally positive affect towards performing health assessments (i.e. satisfaction [33, 34, 47], eagerness [32, 47, 48, 50]. (*n*=6)
	Level of comfort with how patient perceives health assessments	Fear of inadvertently stigmatising the patient [32, 42] (*n*=2)	
**Domain 14: Behavioural Regulation (*n*=5)** Anything aimed at managing or changing objectively observed or measured actions.	Organisational factors limiting/aiding health assessment delivery	Organisational and/or logistical issues identified such as troubles with coordination of all parties [30] and issues with the primary care interface [50] (*n*=2)	Enacting, or intent to enact, changes perceived to promote the uptake of health assessments (i.e. dedicated intellectual disability lead [36, 50] and automatic reminders [42]. (*n*=3)
	Importance of seeking feedback from patients		Seeking feedback from patients [33, 42, 50] (*n*=3)

*n =* frequency as expressed by number of publications

#### Domain 1: Knowledge

Factors identified within 13 publications corresponded to the knowledge domain. In the context of this review, this domain encompasses the awareness, or lack thereof, of vital information regarding people with intellectual disability and health assessments. Some practitioners expressed a lack of awareness regarding the adverse health outcomes experienced by people with intellectual disability [12, 32, 48], a lack of understanding about the assessments themselves [12, 31, 36] and unfamiliarity with their proven health benefits [30, 31, 33, 45, 46, 50]. Although some GPs questioned the need for any screening at all in this patient group [45], others were aware of [12, 30, 43, 44], and acknowledged the existence [31, 45] and benefits of, health assessments [30, 34]. More specific barriers included a lack of knowledge regarding precise codes in clinical information systems to identify patients with intellectual disability [32, 50], and of evidence-based preventive care guidelines [31].

#### Domain 2: Skills

Sixteen publications identified factors corresponding to the skills domain, which in the context of this review refers to practitioners’ perspectives about possessing the training and skills required to perform the health assessments. Communication difficulties as a primary obstacle in conducting the health assessments were identified in six publications [30, 32, 34, 41, 48]. For example, practitioners may rely on support workers to communicate with the patient, which has the potential for diminishing the patient’s autonomy and ability to communicate effectively [40]. Conversely, this same publication suggested that interacting directly with the patient establishes both respect for, and empowerment of, the patient. One practice attempted to overcome barriers to communication by assigning all contact with patients to the member of their staff who had the most skills in, and comfort with, communicating with people with intellectual disability [42].

Other barriers mapped included both inadequate exposure to people with intellectual disability, and not enough relevant curriculum content throughout medical school as well as a lack of advanced training in this area [12, 31, 32, 34, 45, 47, 48]. Practitioners also recommended further education on the delivery of care to people with intellectual disability [31–33, 38, 44, 47, 50], and on how to conduct their health assessments [33, 34, 36]. There were a number of GPs who expressed the belief that all patients should be treated the same, which simply highlights the lack of training about the need for reasonable adjustments for people with intellectual disability and targeted interventions to ensure access to care [12, 48, 50].

#### Domain 3: Social/professional role and identity

Factors identified within 10 publications corresponded to the social/professional role and identity domain, which in the context of this review covers the recognition that it is the practitioners’ responsibility to conduct health assessments. While most GPs acknowledged their responsibility to provide medical care to people with intellectual disability, some did not feel that it was their responsibility to undertake a yearly health assessment [37, 43, 45]. Others sought further clarity about the role before committing, as they felt out of their depth [30, 34]. Conversely, several practitioners acknowledged that since people with intellectual disability live in the community, the initiation and management of medical care falls within the remit of general practice [31–33, 37, 45, 47, 50]. There were contradictory views regarding whose role it was to follow up any abnormal findings or referrals required as part of the health assessment. Some practitioners felt themselves to be responsible in ensuring that these plans are followed up and monitored [50], whereas others were confused as to whose role this was [47].

#### Domain 4: Beliefs about capabilities

Factors identified within seven publications corresponded to the beliefs about capabilities domain, which in the context of this review encompasses practitioners’ level of confidence in their ability to conduct health assessments. Practitioners at times felt unprepared, incompetent and/or lacked confidence in their ability to perform all aspects of the health assessments, thereby creating a barrier to their implementation [12, 32, 39, 46]. However, as identified in four publications, some practitioners felt comfortable with caring for people with intellectual disability [34] and believed themselves to be capable of providing adequate care without having a special interest in the patient population [12, 31, 47].

#### Domain 5: Optimism

Factors identified within nine publications corresponded to the optimism domain, which in the context of this review refers to the general belief that health assessments are worthwhile, without specifying any expected outcomes. Barriers to implementing the health assessments were identified in six publications, specifically practitioners’ scepticism about the value of screening [31, 34, 45, 46, 48] and their inability to perceive any associated benefits from providing the assessments [30]. Conversely, a sense of optimism among practitioners that assessments were beneficial for patients was identified in seven publications [12, 30, 31, 33, 34, 47, 48].

#### Domain 6: Beliefs about consequences

Factors identified within 10 publications corresponded to the beliefs about consequences domain, which in the context of this review relates to understanding the potential outcomes of providing health assessments. The majority of publications identified the provision of health assessments as a facilitating factor both for practitioners [12, 32, 47] and for support workers [30] to gain further knowledge on how best to care for patients with intellectual disability. The assessments were also seen as a means of building collaboration between the parties involved [44, 47], and of further developing the practitioner–patient relationship through enhanced continuity of care [32, 47, 48]. Furthermore, there was a common belief among practitioners that assessments specifically lead to an improvement in health outcomes for those patients who utilise them [30, 32, 44, 45, 47, 48, 50]. However, a perception that more evidence on the benefits of health assessments was required to support their implementation was identified in three publications [31, 32, 43].

#### Domain 7: Reinforcement

Factors identified within seven publications corresponded to the reinforcement domain, which in the context of this review looks at the incentives needed to influence the implementation of health assessments. Five publications indicated that practitioners believed there is insufficient financial compensation for the extra time required to prepare for and provide these assessments [12, 32, 38, 47, 48]. However, in two other publications, these sentiments were contradicted, with participants claiming that there was adequate compensation both to implement health assessments [31] and to attend the necessary training [42].

#### Domain 8: Intentions

Factors identified within nine publications corresponded to the intentions domain, which in the context of this review relates to how inclined practitioners are to provide health assessments to people with intellectual disability. Barriers to this included a perceived lack of willingness to do so [47], an explicit admission that the provision of health assessments was not a priority [42] and a general lack of interest in providing care for people with intellectual disability at all [30]. Additionally, some practices had practitioners attempting to conduct the assessment within a 15-min consultation, thereby demonstrating a lack of intent to provide a comprehensive service [36, 48]. Practitioners who were facilitating the implementation of health assessments were driven either by a personal interest [33, 45, 50] or a practice-wide focus [42]. Practices that intended to implement reasonable adjustments—including the offer of home visits [48], weekend clinics [36], greater choice in appointment times, reduced wait times [50] and the provision of Easy Read heath information [39]—were identified in four publications.

#### Domain 9: Goals

A factor identified within one publication corresponded to the goals domain, which indicated that a practice had set a specific goal of providing health assessments to 75% of its patients with intellectual disability within an 18-month period [42]. This facilitating factor demonstrated a commitment to the goal of promoting the delivery of health assessments and to improving the quality of care to people with intellectual disability.

#### Domain 10: Memory, attention and decision processes

Factors identified within seven publications corresponded to the memory, attention and decision processes domain. In the context of this review, this domain relates to the ability to remember, or to pay attention to, the relevant information needed to make informed decisions relating to health assessments. Barriers identified in seven publications were associated with actually identifying people with intellectual disability due to the lack of a sufficient pre-existing registry or list of eligible patients on clinical information systems [32, 33, 36, 42, 43, 48, 50]. One of the publications described a practice that utilised an alert system to inform practitioners about upcoming health assessments. Timely reminders such as this are an excellent mechanism to enhance memory and attention [42].

#### Domain 11: Environmental context and resources

Factors identified within 18 publications corresponded to the environmental context and resources domain. In the context of this review, this domain refers to the availability of the resources needed to encourage or discourage the implementation of health assessments. Concerns were raised about the ability of support workers and advocates to contribute effectively to the assessment process due to a lack of clarity about their roles [30], their unfamiliarity with the patients [31] and even that their involvement could result in disempowering the patients [40] or practitioner [41]. However, the important role that support workers play, both in making patients feel comfortable and in encouraging their acceptance of recommended health interventions, was also recognised [31, 33, 40, 41, 45, 48, 50]. Some practitioners reported that a lack of support workers [30, 38], allied health staff or specialist service providers [31, 32, 37, 48] posed a barrier to conducting health assessments. These professionals were valued for their ability to enhance the process and reduce the time required to perform health assessments [31–33, 37, 43, 45]. There was a suggestion from several GPs that physicians who specialise in treating patients with intellectual disability could aid in the identification of patients requiring assessment [32].

A general lack of resources and inadequate time to support the implementation of health assessments were other barriers indicated by practitioners, as assessments not only take longer than standard consultations but also require additional preparation and training [12, 30–32, 34, 38, 41, 42, 47, 48]. Patient-related barriers identified as acting as a deterrent included the perceived lack of demand for health assessments from people with intellectual disability [46], their limited access to general practice [30], practitioners’ difficulties in contacting patients [48] and the need for longer appointments [42]. Easy access to patient histories [31, 34] and to health assessment template scripts [30, 44, 47, 48], along with electronic compatibility of these templates with existing information systems, were identified as facilitators [42, 47].

#### Domain 12: Social influence

Factors identified within five publications corresponded to the social influence domain, all of which were mapped as facilitators. In the context of this review, this domain relates to interpersonal processes and relationships that influence the implementation of health assessments, such as the encouragement received from colleagues who shared good practices and provided positive reinforcement [50]. Additional support for practices to conduct the health assessments came from stakeholder groups [37, 47] and communities [31, 44] and was also mapped as a motivating factor in their implementation.

#### Domain 13: Emotion

Factors identified within 12 publications corresponded to the emotion domain, which in the context of this review encompasses the complex feelings and attitudes of practitioners regarding the provision of health assessments. Emotions coded as barriers were identified in nine of these publications with burn-out, the most commonly mentioned, appearing in six [34, 37, 38, 43, 45, 46]. Practitioners with an already high workload expressed concerns about feeling overwhelmed by the additional work required to provide health assessments. Conversely, within six publications facilitating factors were identified with the most frequently mentioned being eagerness to perform assessments [32, 47, 48, 50] and satisfaction with the care that practitioners were able to provide [33, 34, 47]. Other less commonly identified barriers included anxiety about performing health assessments [33] and an aversion to completing the checklists [32], along with the fear of stigmatising patients, particularly if they had not yet received a formal diagnosis of intellectual disability [32, 42].

#### Domain 14: Behavioural regulation

Factors identified within five publications corresponded to the behavioural regulation domain, which in the context of this review refers to the self-monitoring and management of implementation strategies that will continuously improve the health assessment process. Barriers relating to organisational factors were identified in two publications. One highlighted the coordination issues that arise from the regular scheduling of these periodic assessments [30], while the other described a practice’s difficulties in scheduling patients due to the limitations of its clinical information system [50]. Facilitating factors identified in two publications included the recruitment of a coordinator to a practice to handle the organisation and uptake of health assessments [36, 50] and a proposal by another practice to automate its computer system to prompt staff when a patient was due for their next assessment [42]. Other facilitating factors mapped related to whether practices actively sought [33, 42] or responded to feedback from patients and their families. Feedback that was thought to improve the implementation of health assessments was identified in three publications [50].

## Discussion

This scoping review identified a range of barriers and facilitators that influence the implementation of health assessments in primary care for people with intellectual disability. These were mapped to each of the 14 TDF domains. Potential barriers and facilitators were identified within each domain. The most commonly identified barriers were a lack of awareness regarding the availability and advantages of health assessments specifically targeting individuals with intellectual disability, and concerns about a perceived deficiency in the training and experience of the health care professionals conducting these assessments. Time constraints, lack of availability of staff to support health assessment processes, and practitioner burnout given already high workloads also emerged as barriers. A further barrier to implementation was the inability of many practices to identify the records of patients with intellectual disability in primary care clinical information systems.

Conversely, several facilitators were identified and mapped. Primary care professionals recognised their role in providing health assessments to people with intellectual disability and an eagerness to provide preventive health care. Also identified was a belief in the overall effectiveness of assessments in improving health outcomes, and the potential for these health assessments to facilitate care coordination among practitioners, support personnel and others. Access to resources such as health assessment template scripts, complemented by the electronic compatibility of these templates with existing information systems, was highlighted as pivotal in supporting successful implementation.

The most frequently mapped TDF domains were as follows: (a) environmental context and resources, (b) skills, (c) knowledge and (d) emotion. The predominance of the environmental context and resources domain identified in this review is consistent with other studies that have utilised the TDF to assess barriers and facilitators to accessing preventive health care [22, 23, 51]. It is also in line with contemporary evidence about the importance of taking a systems perspective when implementing interventions [52]. Under-reported in our review were the domains of (a) goals, (b) behavioural regulation and (c) social influences. Similarly, Atkins and colleagues [23] undertook a systematic review using the TDF to examine the uptake of health assessments for people aged 40–74 years in the UK and found a paucity of reporting of the TDF domains related to goals and behavioural regulation. Interestingly, our study differed with Atkins and colleagues [23] in that we found a deficit in the reporting of barriers and facilitators related to social influences whereas they did not. There is a need for further inquiry into these three TDF domains to ensure that primary care practices have a nuanced understanding of the barriers and facilitators to implementation of health assessments for people with an intellectual disability.

Consistent with our review, common findings across studies that have used the TDF to examine uptake of health assessments for other targeted population groups have included the perception that practitioners are inadequately trained in the delivery of comprehensive health assessments [22, 23] the belief that screening and preventive care should be performed by specialists in the respective patient population’s field [22, 23] and a perceived lack of knowledge about relevant health information relating to the patient group [21, 22].

A key finding in our review was that many practitioners identified a lack of skills, knowledge and confidence in providing preventive health care to people with intellectual disability. This is unsurprising given that audits of medical and nursing curricula in Australia revealed that, on average, less than 6 h of teaching time was devoted to intellectual disability throughout any of the degree programmes, with the majority of nursing schools providing none [53, 54]. The Royal Commission into Violence, Abuse, Neglect and Exploitation of People with Disability, established in 2019, found that Australian health professionals often do not have the knowledge, skills and attitudes needed for addressing the health needs of people with intellectual disability [55, 56]. However, research indicates that those health professionals who have received training in disability-related knowledge and communication skills feel more positive and confident in delivering care to those with disability [57].

Although this review included international literature with no date limits applied, there were only 21 publications, derived from 20 studies that met the eligibility criteria. This limited amount of literature also only comes from four high-income countries—the UK, Australia, Canada and the Netherlands. This is likely to be because these countries have policy settings related to the implementation of structured annual health assessments for people with intellectual disability as part of routine practice in primary care, as well as the resources to investigate their impact. For example, in the UK and Australia, there are specific policy directives to strengthen the uptake of health assessments, such as Australia’s National Roadmap to Improve Health Outcomes for People with Intellectual Disability [5] and the UK’s Direct Enhanced Service [6].

The need to improve health outcomes for people with intellectual disability is gaining increasing attention. However, even though interventions or actions designed to address known barriers to quality care are more likely to produce change, there have been few interventions based on a systematic assessment of barriers [58, 59]. As such, this review provides a foundation for future primary research regarding relevant behavioural change interventions [60]. In addition, there is a need for more qualitative research that examines the perceptions of primary care practitioners regarding the implementation barriers and facilitators to health assessments in general and that includes the views of primary care practitioners who are not currently undertaking health assessments.

### Strengths and limitations

The strengths of our review are as follows: 1) a published a priori protocol; and 2) the rigour of having two reviewers independently conducting screening, full text review and data extraction. Review limitations include: 1) the risk of language bias as only publications in English were included; 2) potentially missing relevant evidence as we excluded grey literature and theses; 3) possible selection bias as more than half of the publications involved practitioners who were already implementing health assessments and so would potentially be more motivated to conduct them; 4) all publications were from high-income countries—potentially reflecting where the policy initiatives have driven related investigation.

The comprehensiveness of our review is contingent upon the scope of the incorporated studies, and these have not all taken a comprehensive approach to investigating barriers and facilitators that hinder or support the implementation of health assessments. Consequently, the insights only present a partial picture of influences on behaviours. To clarify, when a TDF domain is indicated as irrelevant to a certain behaviour, it could stem from the fact that no investigation into the barriers and facilitators related to that domain was conducted in the study, rather than from concrete evidence suggesting its irrelevance. A further limitation is that our coding of the barriers and enablers to the most predominant domain does not account for potential relevance of barriers and enablers across domains.

## Conclusions

Using a well-established theory-based framework, this scoping review provides a synthesis of the current literature describing barriers and enablers that impact on the implementation of comprehensive health assessments for people with intellectual disability in the primary care setting. Further inquiry into the TDF domains of (a) goals, (b) behavioural regulations and (c) social influences may be warranted to ensure a comprehensive understanding of what drives and constrains the implementation of health assessments for people with intellectual disability in primary care. These insights provide a foundation for future research to improve the delivery and accessibility of preventive care for people with intellectual disability.

### Supplementary Information

**Supplementary Material 1.**

## Data Availability

Available on request, by contacting the corresponding author.

## Abbreviations

PRISMA-ScR: Preferred Reporting Items for Systematic Reviews and Meta-Analyses Extension for Scoping Reviews
TDF: Theoretical Domains Framework
