# Supplementary material for: Identifying barriers and facilitators to primary care practitioners implementing health assessments for people with intellectual disability: a Theoretical Domains Framework-informed scoping review
Source: Implement Sci Commun. 2024 Apr 16;5:39. doi: 10.1186/s43058-024-00579-8 (PMC11020327; doi:10.1186/s43058-024-00579-8)
Supplement: Supplementary file 1 — Supplementary Material 1. [file 43058_2024_579_MOESM1_ESM.docx]

**Additional Files**

**Additional File 1: Search Strategy**

**Database 1: Medline (OVID-SP) – Coverage 1946 – Present**

1 Exp Intellectual Disability/ OR Fragile X Syndrome/ OR Rett Syndrome/ OR Angelman Syndrome/ OR Developmental Disabilities/ OR Learning Disabilities/

2 (Intellectual Disabilit* OR Intellectual Disorder* OR Cri-Du-Chat Syndrome* OR Down Syndrome* OR De Lange Syndrome* OR Mental Retardation* OR Rubinstein?Taybi Syndrome* OR Trisomy 13 Syndrome* OR WAGR Syndrome* OR Williams Syndrome* OR Prader?Willi Syndrome* OR Genetic Disorder* OR Fragile X* OR Rett* OR Angelman* OR Cat Cry Syndrome* OR Happy Puppet Syndrome* OR Developmental Disabilit* OR  Learning Disabilit* OR Mental Handicap* OR Mental Deficien* OR Mental Retard*).mp.

3  1 OR 2

4 Exp Primary Health Care/  OR Preventive Medicine/ OR Exp General Practice/ OR Health Services/ OR Health Services OR Persons With Disabilities/ OR Health Services, Indigenous/ OR Preventive Health Services/ OR Community Health Services/ OR Nurse Practitioners/ OR Family Nurse Practitioners/ OR Primary Care Nursing/ OR Physicians, Family/

5      (Primary* Care*OR Preventative Medicine*OR General Practice* OR Family Practice* OR GP Service* OR Family Doctor* OR Family Medicine* OR Health Service* OR "Health Services OR Persons With Disabilit*" OR Indigenous Health Service* OR Preventive Health Service* Community* Health Service* OR Aboriginal Health Service* OR Nurse Practitioner* OR Family Nurse Practitioner* OR Primary Care Nurs* OR Family Physician*).mp.

6      4 OR 5

7       (Health Check* OR Health Assessment* OR Preventive Health Check* OR Health Screen*).mp.

8       1 AND 6 AND 7

**Database 2: Embase (OVID-SP) Coverage 1947-Present**

1 Intellectual Impairment/ OR Fragile X Syndrome/ OR Rett Syndrome OR Happy Puppet Syndrome/ OR Cat Cry Syndrome/ OR Down Syndrome/ OR De Lange Syndrome/ OR Mental Deficiency/ OR Rubinstein Syndrome/ OR Trisomy 13/ OR WAGR Syndrome/ OR Williams Beuren Syndrome/ OR Prader Willi Syndrome/ OR Fragile X Syndrome/ OR Genetic Disorder/ OR Rett Syndrome/ OR Developmental Disorder/ OR Learning Disorder/ OR Mental Retardation Malformation Syndrome/

2 (Intellectual Disabilit* OR Intellectual Disorder* OR Cri-Du-Chat Syndrome* OR Down Syndrome* OR De Lange Syndrome* OR Mental Retardation* OR Rubinstein?Taybi Syndrome* OR Trisomy 13 Syndrome* OR WAGR Syndrome* OR Williams Syndrome* OR Prader?Willi Syndrome* OR Genetic Disorder* OR Fragile X* OR Rett* OR Angelman* OR Cat Cry Syndrome* OR Happy Puppet Syndrome* OR Developmental Disabilit* OR Developmental Disorder* OR Learning Disabilit* OR Learning Disorder* OR Mental Handicap* OR Mental Deficien* OR Mental Retard*).mp

3 1 OR 2

4 Primary Health Care/ OR Preventive Medicine/ OR General Practice/ OR Health Service/ OR Indigenous Health Care/ OR Preventive Health Service/ OR Community Care/ OR Nurse Practitioner/ OR Family Nurse Practitioner/ OR Primary Health Care/ OR General Practitioner/

5 Primary* Care* OR Preventative Medicine OR General Practice* OR Family Practice* OR GP Service* OR Family Doctor* OR Family Medicine* OR Health Service* OR "Health Services For Persons With Disabilit*" OR Indigenous Health Service* OR Preventive Health Service* OR Community* Health Service* OR Aboriginal Health Service* OR Nurse Practitioner* OR Family Nurse Practitioner* OR Primary Care Nurs* OR Family Physician*

6 4 OR 6

7 (Health Check* OR Health Assessment* OR Preventive Health Check* OR Health Screen*).mp.

8 3 AND 6 AND 7

**Database 3: APA Psycinfo (OVID-SP) Coverage 1806 – current**

1 Exp Intellectual Development Disorder/ OR Crying Cat Syndrome/ OR Down's Syndrome/ OR Fragile X Syndrome/ OR Williams Syndrome/ OR Rett Syndrome/ OR Developmental Disabilities/ OR Mental Disorders/ OR Learning Disabilities/

2 (Intellectual Disabilit* OR Intellectual Disorder* OR Cri-Du-Chat Syndrome* OR Down Syndrome* OR De Lange Syndrome* OR Mental Retardation* OR Rubinstein?Taybi Syndrome* OR Trisomy 13 Syndrome* OR WAGR Syndrome* OR Williams Syndrome* OR Prader?Willi Syndrome* OR Genetic Disorder* OR Fragile X* OR Rett* OR Angelman* OR Cat Cry Syndrome* OR Happy Puppet Syndrome* Developmental Disabilit* OR Learning Disabilit* OR Mental Handicap* OR Mental Deficien* OR Mental Retard* OR Mental Disorder*).mp

3 1 OR 2

4 Primary Health Care/ OR Preventive Health Services/ OR General Practitioners/ OR Health Care Services/ OR Preventive Health Services/ OR Family Physicians/

5 (Primary* Care* OR Preventative Medicine* OR General Practice* OR Family Practice* OR GP Service* OR Family Doctor* OR Family Medicine* OR Health Service* OR "Health Services For Persons With Disabilit*" OR Indigenous Health Service* OR Preventive Health Service* OR Community* Health Service* OR Aboriginal Health Service* OR Nurse Practitioner* OR Family Nurse Practitioner* OR Primary Care Nurs* Family Physician*).mp.

6 4 OR 5

7 (Health Check* OR Health Assessment* OR Preventive Health Check* OR Health Screen*).mp

8 3 AND 6 AND 7

**Database 4: CINAHL (EBSCO Host) – Coverage 1982 – Present**

S1 (MH “Intellectual Disability+”) OR (MH “Fragile X Syndrome”) OR (MH “Angelman Syndrome”) OR (MH "Developmental Disabilities") OR (MH “Intellectual Disability+”)

S2 "Intellectual Disabilit*" OR "Intellectual Disorder*" OR "Cri-Du-Chat Syndrome*" OR "Down Syndrome*" OR "De Lange Syndrome*" OR "Mental Retardation*" OR "Rubinstein?Taybi Syndrome*" OR "Trisomy 13 Syndrome*" OR "WAGR Syndrome*" OR "Williams Syndrome*" OR "Prader?Willi Syndrome*" OR "Genetic Disorder*" OR "Fragile X*" OR Rett* OR Angelman* OR "Cat Cry Syndrome*" OR "Happy Puppet Syndrome*" OR "Developmental Disabilit*" OR “Learning Disability*” OR "Mental Handicap*" OR "Mental Deficien*" OR "Mental Retard*”

S3 S1 OR S2

S4 (MH “Primary Health Care”) OR (MH "Preventive Health Care+") OR (MH "Family Practice") OR (MH "Health Services For Persons With Disabilities") OR (MH "Health Services, Indigenous") OR (MH "Health Services, Indigenous") OR (MH "Community Health Services+") OR (MH "Community Mental Health Services+") OR (MH "Health Services, Indigenous") OR (MH “Nurse Practitioners+”) OR (MH “Family Nurse Practitioners”) OR (MH "Physicians, Family")

S5 “Primary* Care*” OR "Preventive Medicine*” OR "General Practice*" OR "Family Practice*" OR "GP Service*" OR "Family Doctor*" OR "Family Medicine*" OR "Health Service*" OR "Health Services For Persons With Disabilit*" OR "Indigenous Health Service*" OR "Preventive Health Service*" OR "Community* Health Service*" OR "Aboriginal Health Service*" OR "Indigenous Health Service*" OR "Nurse Practitioner*" OR "Family Nurse Practitioner*" OR “Primary Care Nurs*” OR "Family Physician*"

S6 S4 OR S5

S7 “Health Check*” OR “Health Assessment*” OR “Preventive Health Check*” OR TI "Health Screen*" OR AB "Health Screen*"

S8 S3 AND S6 AND S5

**Database 5: Scopus (Elsevier) Coverage 1996 – Present**

( TITLE-ABS-KEY ( "Intellectual Disabilit*" OR "Intellectual Disorder*" OR "Cri-Du-Chat Syndrome*" OR "Down Syndrome*" OR "De Lange Syndrome*" OR "Mental Retardation*" OR "Rubinstein?Taybi Syndrome*" OR "Trisomy 13 Syndrome*" OR "WAGR Syndrome*" OR "Williams Syndrome*" OR "Prader?Willi Syndrome*" OR "Genetic Disorder*" OR "Fragile X*" OR Rett* OR Angelman* OR "Developmental Disabilit*" OR "Learning Disabilit*" OR "Mental Handicap*" OR "Mental Deficien*" OR "Mental Retard*" OR "Cat Cry Syndrome*" OR "Happy Puppet Syndrome*" )

AND TITLE-ABS-KEY ( "Primary* Care*" OR "Preventive Medicine*" OR "General Practice*" OR "Family Practice*" OR "GP Service*" OR "Family Doctor*" OR "Family Medicine*" OR "Health Service*" OR "Health Services For Persons With Disabilit*" OR "Indigenous Health Service*" OR "Preventive Health Service*" OR "Aboriginal Health Service*" OR "Indigenous Health Service*" OR "Nurse Practitioner*" OR "Family Nurse Practitioner*" OR "Primary Care Nurs*" OR "Family Physician*" )

AND TITLE-ABS-KEY ( "Health Check*" OR "Health Assessment*" OR "Preventive Health Check*" OR "Health Screen*" ) )

**Database 6: Web Of Science (Clarivate) Coverage 1900 – Present**

1 "Intellectual Disabilit*" OR "Intellectual Disorder*" OR "Cri-Du-Chat Syndrome*" OR "Down Syndrome*" OR "De Lange Syndrome*" OR "Mental Retardation*" OR "Rubinstein?Taybi Syndrome*" OR "Trisomy 13 Syndrome*" OR "WAGR Syndrome*" OR "Williams Syndrome*" OR "Prader?Willi Syndrome*" OR "Genetic Disorder*" OR "Fragile X*" OR Rett* OR Angelman* OR "Developmental Disabilit*" OR "Learning Disabilit*" OR "Mental Handicap*" OR "Mental Deficien*" OR "Mental Retard*" OR "Cat Cry Syndrome*" OR "Happy Puppet Syndrome*" (Topic)

**2** "Primary* Care*" OR "Preventive Medicine*" OR "General Practice*" OR "Family Practice*" OR "GP Service*" OR "Family Doctor*" OR "Family Medicine*" OR "Health Service*" OR "Health Services For Persons With Disabilit*" OR "Indigenous Health Service*" OR "Preventive Health Service*" OR "Aboriginal Health Service*" OR "Indigenous Health Service*" OR "Nurse Practitioner*" OR "Family Nurse Practitioner*" OR "Primary Care Nurs*" OR "Family Physician*" (Topic)

**3** "Primary* Care*" OR "Preventive Medicine*" OR "General Practice*" OR "Family Practice*" OR "GP Service*" OR "Family Doctor*" OR "Family Medicine*" OR "Health Service*" OR "Health Services For Persons With Disabilit*" OR "Indigenous Health Service*" OR "Preventive Health Service*" OR "Aboriginal Health Service*" OR "Indigenous Health Service*" OR "Nurse Practitioner*" OR "Family Nurse Practitioner*" OR "Primary Care Nurs*" OR "Family Physician*" (Topic)

**4** "Health Check*" OR "Health Assessment*" OR "Preventive Health Check*" OR “Health Screen*” (Topic)

**5** #1 AND #3 AND #4

**Additional File 2: Data Extraction Template**

*Table 2.1 Data Extraction Template*

| Study Characteristics | Author(S) | Free Text |
| --- | --- | --- |
|  | Year Of Publication | Free Text |
|  | Country The Study Was Conducted (Include Province OR State If Available) | Free Text |
|  | Author Stated Study Aims And/OR Objectives | Free Text, As Stated By Study Authors, Draw On Both Abstract And Introduction |
|  | Study Design | Categories (Qualitative, Quantitative, Mixed Methods) |
|  | Methods Used | Categories (Focus Groups, Interviews, Survey, Direct Observation, Case Reports, Document Review, Other) |
|  | Study Setting | Free Text |
|  | Rurality Of Study Setting (If Stated | Free Text |
|  | Participant Number And Type | Free Text |
| **Domains** | **Theoretical Domains Framework (TDF)** | **Categories** |
| Individual TDF Domains | Knowledge | Free Text |
|  | Skills | Free Text |
|  | Social/Professional Role and Identity | Free Text |
|  | Beliefs About Capabilities | Free Text |
|  | Optimism | Free Text |
|  | Beliefs About Consequences | Free Text |
|  | Reinforcement | Free Text |
|  | Intentions | Free Text |
|  | Goals | Free Text |
|  | Memory, Attention and Decision Processes | Free Text |
|  | Environmental Context and Resources | Free Text |
|  | Social Influences | Free Text |
|  | Emotion | Free Text |
|  | Behavioural Regulation | Free Text |
|  | New Concept | Free Text |
